# Effect of influenza and pneumococcal vaccines in elderly persons in years of low influenza activity

**DOI:** 10.1186/1743-422X-5-52

**Published:** 2008-04-28

**Authors:** Brith Christenson, Karlis Pauksen, Staffan PE Sylvan

**Affiliations:** 1 Department of Communicable Disease Control and prevention, Uppsala County Counci, Dag Hammarskjolds vag 17, SE-751-85 Uppsala, Sweden; 2 Department of Medical sciences, Infectious Diseases, Uppsala University Hospital Uppsala, Sweden

## Abstract

**Background:**

The present prospective study was conducted from 2003–2005, among all individuals 65 years and older in Uppsala County, a region with 300 000 inhabitants situated close to the Stockholm urban area.

The objective of this study was to assess the preventive effect of influenza and pneumococcal vaccination in reducing hospitalisation and length of hospital stay (LOHS) even during periods of low influenza activity. The specificity of the apparent vaccine associations were evaluated in relation to the influenza seasons.

**Results:**

In 2003, the total study population was 41,059, of which 12,907 (31%) received influenza vaccine of these, 4,447 (11%) were administered the pneumococcal vaccine. In 2004, 14,799 (34%) individuals received the influenza vaccine and 8,843 (21%) the pneumococcal vaccine and in 2005 16,926 (39%) individuals were given the influenza vaccine and 12,340 (28%) the pneumococcal vaccine.

Our findings indicated that 35% of the vaccinated cohort belonged to a medical risk category (mainly those persons that received the pneumococcal vaccine). Data on hospitalisation and mortality during the 3-year period were obtained from the administrative database of the Uppsala county council.

During the influenza seasons, reduction of hospital admissions and significantly shorter in-hospital stay for influenza was observed in the vaccinated cohort (below 80 years of age). For individuals who also had received the pneumococcal vaccine, a significant reduction of hospital admissions and of in-hospital stay was observed for invasive pneumococcal disease and for pneumococcal pneumonia. Effectiveness was observed for cardiac failure even in persons that also had received the pneumococcal vaccine, despite that the pneumococcal vaccinated mainly belonged to a medical risk category. Reduction of death from all causes was observed during the influenza season of 2004, in the 75–84-year old age group and in all age-groups during the influenza season 2005.

**Conclusion:**

The present study confirmed the additive effect of the two vaccines in the elderly, which was associated with a reduced risk in hospitalisation and a reduction in mean LOHS in seasons with low influenza activity.

## Background

The administration of influenza and pneumococcal vaccines has been limited in many European countries. Only 4–8% of all persons in Sweden, received influenza vaccine up to 1995. After 1997, however, the influenza vaccine distribution increased considerably in Sweden as in many other countries [1,2]. An annual influenza vaccination among persons aged 65 years and older has been recommended since 1997 and, according to the Swedish National Board of Health and Welfare, pneumococcal vaccination should also be considered for this age group. The administration of both vaccines is also recommended in persons with lung and heart problems.

The vaccination rate increased in the Stockholm area from 40% in 1998 to 48% in 1999 [3]. Earlier, costs of influenza and pneumococcal vaccines have not been reimbursed in Sweden. After 2000, however, several counties in Sweden, including Stockholm County, introduced reimbursement for vaccination, which appears to be associated with greater vaccine use. After the introduction of the reimbursement policy, the vaccination rate has increased to about 60% in the Stockholm County. On the other hand, influenza vaccination is not reimbursed in Uppsala County; despite this policy, the vaccination rate in Uppsala County increased from 31% in 2003 to 39% in 2005.

The benefit of annual influenza vaccination has been documented in several case-control and retrospective cohort studies [4-10]. Influenza vaccination has been reported to reduce the need for hospitalisation in chronic respiratory conditions and heart failure [11]. It has also been found to reduce hospitalisation in cardiac disease and stroke in elderly patients [12]. The effectiveness of influenza vaccination and mortality in elderly persons, however, has more recently been questioned, where it has been explained by an unrecognised selection bias in cohort studies [13,14].

Retrospective studies have shown that pneumococcal vaccine is efficacious against pneumonia and reduces hospitalisation and death that is due to pneumonia in elderly people with chronic lung disease [15]. An additive effect of influenza and pneumococcal vaccination in elderly persons with chronic lung disease or cardiac failure has also been demonstrated [16]. The additive effect of both vaccines has been supported by prospective intervention studies preformed in1999 and 2000 in Stockholm County [3,17,18].

This paper concerned the efficacy and benefit of influenza vaccination in an elderly population during periods with low influenza activity. A further concern was to study the preventive effect of the pneumococcal vaccine. In the present study we found that vaccination of elderly persons was effective in preventing hospitalisation and in mean length of hospital stay and also in reduction of mortality that were caused by influenza and pneumonia.

## Results

**Figure 1:**
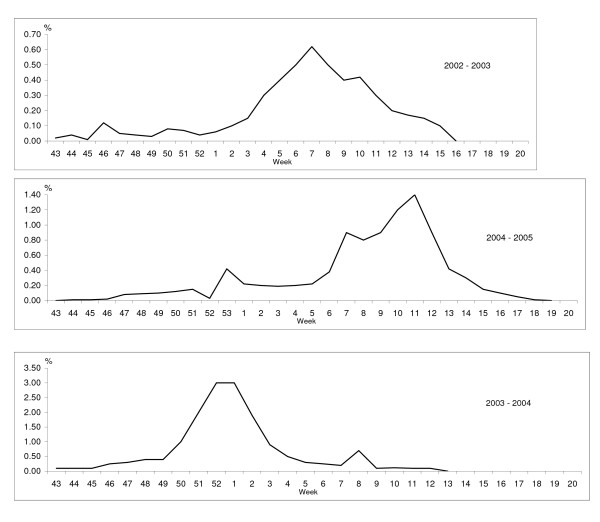
Proportion (%) of influenza-like illness out of a total number of patient visits in the sentinel system.

The influenza activity was low in Uppsala region as in the rest of Sweden during the three influenza seasons (i.e.2003–2005) (Figure 1). The lowest influenza activity was observed in 2003. The influenza season peaked during the middle of February with an incidence of 0.64% among patients who sought the general practitioner that participated in the sentinel influenza campaign. In 2004, the influenza season was short; peaking at 3.1% during the last week of December 2003 and the first week of January 2004. In 2005, the influenza activity occurred around Christmas and peaked in the beginning of March with an incidence of 1.3%.

Table 1 shows the proportion of the study population and the distribution of the vaccinated individuals as a function of age. No difference was found in the proportion of men and women among vaccinated and unvaccinated individuals (data not shown).

**Table 1:** Proportion of vaccinated individuals in different age groups, 2003–2005 in Uppsala County.

	Individuals no			Influenza vaccinated %			Influenza- and pneumococcal vaccinated %		
			
Age groups	≥ 65	65 – 79	≥ 80	≥ 65	65 – 79	≥ 80	≥ 65	65 – 79	≥ 80
2003	41 059	28 177	12 861	31	28	40	11	10	14
2004	43 131	29 743	13 388	34	30	42	21	19	25
2005	43 781	30 270	13 510	39	36	45	28	27	31

The influenza vaccination rate increased during the 3-year study period and was highest in the age group 80 years and older.

The incidence of hospital admissions and of in-hospital stay for influenza varied during the three influenza seasons (December 1 to May 31 for the period 2003–2005). In 2003, no influenza case of an individual below 80 years old in the vaccinated cohort was admitted to hospital. No significant reduction of hospital admissions was observed. However, the in-hospital stay calculated as mean LOHS (days) was significantly shorter for the vaccinated cohorts in 2003 and 2004 (Table 2).

**Table 2:** Incidence and reduction of hospital admissions and in-hospital treatment (days) for influenza with or without respiratory disease per 100 000 in vaccinated and unvaccinated cohorts during the influenza seasons (1 December – 31 May), 2003 – 2005.

		Hospital admission				In-hospital treatment			
			
		Incidence		Reduction		Incidence		Reduction	
					
	Age-group	Vaccinated	Unvaccinated	(95% CI)	*p*	Vaccinated	Unvaccinated	(95% CI)	*p*
2003									
	65 – 79	0	19			0	98		
	≥ 80	39	103	62% (0,08–1,84)	< 0.2	254	826	69% (0,17–0,57)	< 0.001
2004									
	65 – 79	55	63	14% (0,46–1,59)	< 0.8	175	379	54% (0,27–0,80)	< 0.01
	≥ 80	301	297	- 1% (0,53–1,90)	< 0.96	1683	3073	46% (0,43–0,69)	< 0.001
2005									
	65 – 79	83	103	20% (0,36–1,76)	< 0.6	471	540	13% (0,62–1,40)	< 0.4
	≥ 80	441	351	- 21% (0,73–2,2)	< 0.5	2684	2297	- 15% (0,94–1,45)	< 0.2

Concerning invasive pneumococcal disease, the reduction of hospital admissions was 68% (< 005) and for in-hospital stay 40% (<0.001) among persons who had received both the pneumococcal and influenza vaccine as compared with the non-vaccinated cohort and those who had received only the influenza vaccine (data not shown) during the period 2003–2005 (Table 3). For pneumococcal pneumonia, the reduction of hospital admissions was 13% (< 0.8) and the reduction of in-hospital stay was 38% (< 0.001)

**Table 3:** Incidence and reduction of hospital admissions and in-hospital treatment (days) for invasive pneumococcal disease and pneumococcal pneumonia in influenza pneumococcal vaccinated and unvaccinated cohorts, 2003 – 2005.

	Hospital admission			In-hospital treatment		
		
	Incidence	Reduction		Incidence	Reduction	
		(95% CI)	*p*		(95% CI)	*p*
*Invasive pneumococcal disease*						
Vaccinated	12			234		
		68% (0,1–1,06)	< 0.05		40% (0,46–0,78)	< 0.001
Unvaccinated	37			401		
*Pneumococcal pneumonia*						
Vaccinated	59			328		
		13% (0,5–1,5)	< 0.8		38% (0,49 – 0,77)	< 0.001
Unvaccinated	67			532		

For pneumonia overall (Table 4) a reduction of hospital admissions (38%–55%) and of in hospital treatment (13%–49%) was found for individuals who had received the influenza vaccine only during the three influenza seasons. No reduction was found in individuals who had received both vaccines (data not shown).

**Table 4:** Incidence per 100 000 individuals of hospital admissions and in-hospital treatment (days) for pneumonia over all in influenza vaccinated individuals (65 – 79 years) compared with the unvaccinated cohort during the influenza- and non-influenza seasons.

	Hospital admission				In-hospital treatment			
		
*Influenza seasons*	Vaccinated	Reduction (95% CI)	Unvaccinated	*p*	Vaccinated	Reduction (95% CI)	Unvaccinated	*p*
2003	199		388		1548		1781	
		49% (0,26–0,99)		< 0.04		13% (0,68–1,1)		< 0.7
2004	193		310		1408		1982	
		38% (0,28–1,36)		< 0.15		29% (0,53–0,95)		< 0.02
2005	144		319		865		1691	
		55% (0,28–1,36)		< 0.04		49% (0,34–0,78)		< 0.001

*Non-influenza seasons*								
2003	119	49% (0,21–1,20)	231	< 0.2	615	63% (0,26–0,55)	1646	< 0.001
2004	304	- 17% (0,62–2,32)	253	< 0.6	2126	- 31% (1,12–1,85)	1482	< 0.01
2005	252	- 10% (0,60–3,06)	226	< 0.8	1983	- 20% (0,93–1,70)	1583	< 0.2

To estimate the possible protective efficacy for cardiac failure individuals who had received either influenza vaccine or both vaccines were compared with the non-vaccinated cohort during the influenza seasons versus the non-influenza seasons (Table 5). This comparison showed that the persons who had received the influenza vaccine only and those who had received both vaccines had a significantly shorter in-hospital stay during the influenza seasons (except for 2003) when there was low influenza activity and no influenza case was admitted to hospital in the age group 65–79 years.

**Table 5:** In hospital treatment (days) per 100 000 individuals for cardiac failure in influenzavaccinated and influenza- and pneumococcal- vaccinated individuals compared with the unvaccinated cohort.

		Influenza vaccinated			Influenza and pneumococcal			Non-vaccin
				
		Incidence	Reduction (95% CI)	*p*	Incidence	Reduction (95% CI)	*p*	Incidence
2003	Influenza season							
	65 – 79 years	3812	- 25% (1,09–1,52)	< 0.01	4175	- 32% 1,19–1,77)	< 0.001	2914
	Non-influenza season							
	65 – 79 years	6353	- 72% (3,03–4,08)	< 0.001	944	50% (0,35–0,75)	< 0.001	1889
2004	Influenza season							
	65 – 79 years	1537	53% (0,36–0,62)	< 0.001	2491	24% (0,63–0,92)	< 0.005	3265
	Non-influenza season							
	65 – 79 years	4279	- 38% (1,35–1,92)	< 0.001	3051	- 12% (0,74–1,05)	< 0.2	2687
2005	Influenza season							
	65 – 79 years	1658	62% (0,28–0,51)	< 0.01	3046	29% (0,62–0,82)	< 0.001	4215
	Non-influenza season							
	65 – 79 years	360	77% (0,07–0,25)	< 0.001	2536	5% (0,81–1,11)	< 0.7	2647

When looking at death from all causes in relation to age and vaccination status during the influenza seasons versus the non-influenza seasons, 2003–2005, a reduction in overall mortality was noted, during the influenza season of 2004, for the age-group 75–84 years, and in all age groups during the 2005 influenza season (Table 6).

**Table 6:** Deaths from all causes and the reductions of deaths according to age and vaccination status, 2003 – 2005.

Age	65 – 74		75 – 84		≥ 85	
Vaccinated	yes	no	yes	no	yes	no
*2003 Influenza season*						
Deaths/100 000	491	706	2235	2270	7224	7317
Reduction (95% CI) *p*	31% (0,44–1,07) < 0.1		2% (0,79–1,22) < 0.9		1% (0,81–1,20) < 0.9	
*Non-influenza season*						
Deaths/100 000	610	767	2440	2332	6677	7580
Reduction (95% CI) *p*	21% (0,53–1,18) < 0.6		- 5% (0,85–1,28) < 0.9		12% (0,72–1,07) < 0.2	
*2004 Influenza season*						
Deaths/100 000	973	837	2012	2566	8401	8974
Reduction (95% CI) *p*	- 16% (0,85–1,59) < 0.4		22% (0,62–0,97) < 0.03		7% (0,78–1,12) < 0.4	
*Non-influenza season*						
Deaths/100 000	730	646	2028	2214	7667	8171
Reduction (95% CI) *p*	- 12% (0,79–1,65) < 0.5		9% (0,72–1,15) < 0.5		7% (0,77–1,13) < 0.5	
*2005 Influenza season*						
Deaths/100 000	502	882	2144	2829	6979	8882
Reduction (95% CI) *p*	43% (0,40–0,82) < 0.002		25% (0,62–0,92) < 0.01		23% (0,64–0,92) < 0.01	
*Non-influenza season*						
Deaths/100 000	698	813	1761	2188	6942	7676
Reduction (95% CI) *p*	14% (0,61–1,20) < 0.4		20% (0,64–1,0) < 0.06		11% (0,73–1,08) < 0.3	

## Discussion

Consistent with other findings, the present prospective study showed that influenza and pneumococcal vaccinations are beneficial for elderly persons, even during periods of low influenza activity [9,10,13,16,19]. Three prospective influenza and pneumococcal vaccination intervention studies of an elderly population in Stockholm County in 1999 and 2000, with moderate or high influenza activity found significantly lower hospital admissions for influenza, pneumonia and invasive pneumococcal disease in the vaccinated cohort as compared with the non-vaccinated cohort [3,17,18]. Moreover, an additive effect of the two vaccines was demonstrated. It is well established that respiratory viruses predispose to bacterial complications. On average, 50% of patients hospitalised with influenza have bacterial pneumonia though this figure depends on the viral strain circulating. This was particularly the case in the influenza pandemic of 1918 but even the pandemics of 1957 and 1968 revealed that the virus predisposed to bacterial complications [20]. Annual influenza epidemics are known to cause significant morbidity and mortality [21].

An important issue concerns whether the vaccinated and non-vaccinated cohorts were similar in age and in the underlying chronic disorder. Concerning the prospective studies in Stockholm County [3,17,18], a postal inquiry to a random sample of 10,000 elderly persons was undertaken in Stockholm County to characterise possible confounders that might make vaccinated and non-vaccinated cohorts different at baseline on matters of health and demographic data [22]. The studies found that vaccination rates (<0.001) were lower in healthy senior citizens than in elderly individuals with underlying chronic heart or lung disease. The presence of a chronic disease was significantly more common in vaccinated than among non-vaccinated persons. We found that 35% of the vaccinated cohorts in the present study belonged to a medical risk category. Between 11 and 28% of the vaccinated cohorts had also received the pneumococcal vaccine which mainly represented individuals with chronic respiratory and heart conditions.

Thus, these data could indicate that the findings may actually underestimate, rather than overestimate the beneficial effect of influenza and pneumococcal vaccination. This observation is consistent with the results from Nichol et al. [12], who found that vaccinated individuals at baseline were on average, sicker and had higher rates of prior hospitalisation for pneumonia and most co-existing conditions [11]. However, the authors also reported that non-vaccinated persons were more likely to have prior diagnosis of dementia or stroke.

Whether persons vaccinated are healthier than non-vaccinated persons is unclear. A recent study has questioned weather individuals who were vaccinated were healthier than non-vaccinated individuals, possibly biasing estimates of effectiveness upward [23]. However, after correcting for confounding in persons 64 years and older for differences in demographics and underlying health characteristics between vaccinated and non-vaccinated persons, the authors concluded that influenza vaccination reduced the risk of hospitalisation and death that were due to respiratory diseases.

The difference of influenza vaccination reducing hospital admission for influenza, with or without influenza respiratory diseases, did not reach statistical significance This finding can be explained by the fact that the number of patients included was too low for meaningful statistical analysis because of the low influenza activity during the years studied. However, patients requiring hospital admission for influenza, had shorter mean LOHS (except in 2005 for the age-group >80 years) than non-vaccinated patients. The shorter LOHS could indicate that the vaccinated patients had acquired a partial immunity, resulting in a less severe infection. Another explanation, however, could be that the vaccination reduced bacterial complications. This latter explanation is in accord with a recent systematic review of the effectiveness of influenza vaccines in elderly people, where it was noted that the usefulness of vaccines was most evident against complications [13]. It must be emphasised that in the present study only the incidence of hospital care for influenza was investigated, which was very low in the age-group 65–74 years i.e. no data on the protection against influenza *per se *were obtained.

The clinical effectiveness of pneumococcal vaccine in the prevention of pneumococcal pneumonia without bacteraemia has been challenged [24-27]. Yet, the evidence on the protective effect in preventing invasive pneumococcal disease has received some support [28-30]. In the present study, we found a reduction of 68% in hospitalisation (p < 0.05) as well as a significantly shorter mean LOHS (40% p < 0.001) for invasive pneumococcal disease. In pneumococcal pneumonia, the reduction was 13% (p < 0.8) for hospital admissions and 38% (p < 0.001) for in-hospital stay. For pneumonia in general, the reduction of hospitalisation was only observed in persons who had only been given the influenza vaccine. This finding might be consistent with the notion that persons receiving the pneumococcal vaccine were assumed to belong to a medical risk category. The presence of a chronic disease, including heart and lung diseases, was significantly more common in those who had received pneumococcal vaccine [20].

However, a protective effect of the pneumococcal vaccine was observed in cardiac failure (Table 5), which is a finding in accord with other studies [3,16]. Influenza vaccination has been found to reduce the need for hospitalisation in chronic respiratory conditions and heart failure [12,15] and to reduce the risk of primary cardiac arrest [31]. During the influenza seasons of 2004–2005, persons who were administered the influenza vaccine only and those who had received both vaccines had significantly shorter in-hospital stay as compared with the non-vaccinated cohort. During the non-influenza seasons of 2003 and 2004, the persons that were only influenza vaccinated had a significantly longer in-hospital stay than the non-vaccinated cohorts. However, in 2005, the influenza vaccinated had significantly fewer days in hospital than the non-vaccinated cohorts. When compared with the non-vaccinated cohort, the pneumococcal vaccinated had significantly fewer days in hospital in 2003, whereas no difference was noted in hospital stay in the non-influenza seasons of 2004 and 2005.

It is usually assumed that the effectiveness of both these vaccines decline with increasing age [26], an assumption that might explain why the preventive effect was only observed in persons 65–79 years of age.

The efficacy of the influenza vaccine and the all-cause mortality have also been called into question [14]. In a recent review of the effectiveness of influenza vaccination only a moderate, benefit was found in vaccinating elderly persons living in the community [13]. The all-cause mortality reduction was explained by an unrecognised selected bias in cohort studies [14,24]. It was questioned whether an unrecognised sub-population of elderly persons could have led to the overestimation of the benefit of influenza vaccine and the reduction of hospital admission. At the same time, it was concluded that the burden of influenza in the elderly is substantial and even a modest protection of a 30% reduction in influenza- related hospital admissions is beneficial [24].

During periods of at least a moderate level of influenza activity, it is assumed that influenza vaccines are is associated with lower mortality from all causes in the vaccinated cohort as compared with the non-vaccinated cohort. It is further assumed that vaccination is beneficial regarding pre-existing diseases, such as chronic lung or heart diseases. Several studies have documented a reduction in the overall mortality in the influenza-vaccinated cohort as compared with the non-vaccinated cohort[5,11,17-19]. The additive effect of pneumococcal vaccine might contribute to the efficacy in the vaccinated cohort [3]. However, it seems reasonable that a large reduction of mortality of all causes of approximately 50% is exaggerated [14] and cannot be attributed to the vaccine alone. Moreover, there might be a sub-population of non-vaccinated elderly persons with dementia and stroke [12] or other advanced illnesses that contributed to the overestimation.

In the present study in which there was low influenza activity, we found a significant reduction of 25% in mortality during the influenza seasons in ages group 75–84 years (2004) and between 23% and 43% in all age groups (2005).

## Conclusion

The present study confirmed the additive effect of the two vaccines in the elderly even in years with low influenza activity, which was associated with a reduced risk in hospitalisation and a reduction in mean LOHS. Despite that the pneumococcal vaccinated belonged to a risk category effectiveness was observed for invasive pneumococcal disease, pneumococcal pneumonia and cardiac failure. Reduction of death from all causes was observed during the influenza seasons, 2004 and 2005.

## Methods

### Study population

All individuals in Uppsala County aged 65 years and older were requested to take part in a vaccination campaign against influenza and pneumococcal infection.

General practitioners administered most of the vaccinations. At vaccination, the vaccine recipients' name and personal identification code were recorded as well as whether they had been given only influenza vaccine or both vaccines. At the time of vaccination, the vaccine recipients were asked whether they had a lung or cardiovascular disease. The criterion of belonging to a medical risk category was a prescription regarding myocardial or lung diseases.

For analyses, influenza- and pneumonia related diagnoses were identified from 2003–2005 in all individuals 65 years and older that were admitted to hospital in Uppsala County. The vaccination data were matched with discharge diagnoses according to the International Classification of Diseases, 10^th ^revision (ICD-10-CM) and mortality data for all individuals aged 65 years and older in Uppsala County.

In 2003, 12,907 individuals, (31% of the target population) were vaccinated against influenza during the vaccination campaign that took place in the autumn of 2002. In the autumns of 2003 and 2004, 14,799 (34%) and 16,926 (39%) individuals, respectively, were vaccinated against influenza.

Of the target population of 2003, 4,447 (11%) had also received the pneumococcal vaccine, which was 34% of the vaccinated cohort. In 2004, this figure was 8,843 (21%) individuals, (60% of the vaccinated cohort) and in 2005, 12,340 (28%) individuals had also received the pneumococcal vaccine (73% of the vaccinated cohort). An investigation in 2003 of individuals vaccinated against influenza indicated that 35% belonged to a medical risk category defined as chronic lung and/or heart disease.

The influenza vaccines used in 2003 and 2004 were one dose of the recommended trivalent influenza vaccine containing 15 μg of A/New Caledonia/20/99 (H1N1), A/Moscow/10/99 (H3N2) and B/Hong Kong/330/2001-like strain (Batrevac inactivated surface influenza vaccine). In 2005, the trivalent influenza vaccine, A/Fujian/411/2002(H3N2), A/NewCaledonia/20/99(H1N1) and B/Shanghai/361/2002 were used.

The pneumococcal vaccine used was the 23-valent pneumococcal polysaccharide vaccine (Pneumovax from Pasteur-Merieux MSD or Pneumokockvaccin, SBL Vaccin, Stockholm Sweden).

### Endpoints

The primary endpoints were incidence of admissions and number of days in hospital because of influenza (ICD-10; J10.0, J10.8, J11.0, and J11.8), pneumonia (ICD-10; J12–18, J69.0 and A48.1), IPD (ICD-10; A40.3 and G00.1) and cardiac failure (ICD-10; I 500, I 501, I 509) in the vaccinated versus the non-vaccinated cohort. An endpoint diagnosis was accepted irrespective of whether it was on the first or at another place of the discharged diagnoses. However, only one endpoint diagnosis, (the first to appear), per episode of hospital stay was included in the analysis.

The incidence of hospital admissions and the length of hospital stay (LOHS) were compared for the endpoint diagnoses during the three influenza seasons (i.e. December to the end of May) with the non-influenza seasons (June to the end of November).

The vaccinated cohort group included individuals who had received only the influenza vaccine or who had been given both the influenza and the pneumococcal vaccine. Comparisons with the non-vaccinated cohort group were performed against the total vaccinated cohorts or against persons who had received both influenza and pneumococcal vaccines.

### Statistical methods

Differences between vaccinated and non-vaccinated individuals were evaluated using the confidence interval for a proportion and the chi-squared test for categorical variables.

As an estimate of the relative risk, the adjusted odds ratio (OR) was calculated. The reduction in hospital admission and duration of hospital stay were calculated as (1-OR) ×100%.

## Abbreviations

LOHS: Lengths of hospital stay; OR: Odds ratio; CI: Confidence intervals.

## Competing interests

The authors declare that they have no competing interests.

## Authors' contributions

All authors have made substantial contributions to concept, design, acquisition of data, analysis and interpretation and been involved in drafting the manuscript. All authors read and approved the final manuscript.
